# Temporal variation in selection on body length and date of return in a wild population of coho salmon, *Oncorhynchus kisutch*

**DOI:** 10.1186/1471-2148-12-116

**Published:** 2012-07-17

**Authors:** Miyako Kodama, Jeffrey J Hard, Kerry A Naish

**Affiliations:** 1 School of Aquatic and Fishery Sciences, University of Washington, Seattle, WA, 98105, USA; 2 National Marine Fisheries Service, Northwest Fisheries Science Center, Seattle, WA, 98112, USA

**Keywords:** Selection, Temporal variation, Evolution, Environmental variation, Lifetime reproductive success, *Oncorhynchus kisutch*

## Abstract

**Background:**

A number of studies have measured selection in nature to understand how populations adapt to their environment; however, the temporal dynamics of selection are rarely investigated. The aim of this study was to assess the temporal variation in selection by comparing the mode, direction and strength of selection on fitness-related traits between two cohorts of coho salmon (*Oncorhynchus kisutch*). Specifically, we quantified individual reproductive success and examined selection on date of return and body length in a wild population at Big Beef Creek, Washington (USA).

**Results:**

Reproductive success and the mode, direction and strength of selection on date of return and body length differed between two cohorts sampled in 2006 and 2007. Adults of the first brood year had greater success over those of the second. In 2006, disruptive selection favored early and late returning individuals in 2-year-old males, and earlier returning 3-year-old males had higher fitness. No evidence of selection on date of return was detected in females. In 2007, selection on date of return was not observed in males of either age class, but stabilizing selection on date of return was observed in females. No selection on body length was detected in males of both age classes in 2006, and large size was associated with higher fitness in females. In 2007, selection favored larger size in 3-year-old males and intermediate size in females. Correlational selection between date of return and body length was observed only in 2-year-old males in 2006.

**Conclusions:**

We found evidence of selection on body length and date of return to the spawning ground, both of which are important fitness-related traits in salmonid species, but this selection varied over time. Fluctuation in the mode, direction and strength of selection between two cohorts was likely to be due to factors such as changes in precipitation, occurrence of catastrophic events (flooding), the proportion of younger- versus older-maturing males, sex ratio and densities of spawners.

## Background

A number of studies have measured selection in nature in an effort to understand how populations adapt to their environment over time [1,2]. Reviews collating the estimates of selection in natural populations have debated their magnitude, mode and temporal stability [1-7]. However, generalizations have been limited because many of the studies lacked temporal replication, comprised small sample sizes that reduced their statistical power to detect selection, or measured fitness components instead of total fitness [5,7]. Nonetheless, two patterns that do emerge are that phenotypic selection is often strong enough to cause evolutionary changes in relatively few generations, and that directional selection frequently prevails over stabilizing or disruptive selection [4,7]. Several factors may interact to reduce response to directional selection [2], including the fact that selection can vary in strength, direction and mode over time [1,8].

Studies indicate that temporal changes in selection are attributable to variation in environmental and ecological factors, such as changes in climate, sex ratio or density [1,8,9]. Long-term investigation into patterns of selection provides insight into how phenotypic variation in fitness-related traits is maintained, and how populations adapt to variable environmental conditions [1]. Such information also allows predictions on whether and how populations evolve in response to human perturbations, thus facilitating effective conservation and management of exploited species [10-13].

Salmonid fishes provide an ideal study system to study temporal changes in selection. These species are philopatric; in principle, it is possible to sample an entire population at maturity, and the assessment of lifetime reproductive success can be used to accurately measure selection [14,15]. Also, several life history traits have been shown to be under selection in these species [16-21]. For example, several studies have demonstrated that return timing in salmon is often linked to fitness and responds to selection [17-20]. Variability in this trait is typically affected by environmental conditions such as rainfall, temperature or flow regime of their natal river, all of which tend to fluctuate annually [15]. Despite such fluctuations, however, the earlier return of males to the spawning grounds (“protandry”) is commonly reported in salmonids [22]. This behavior may maximize mating opportunities in males, as females may not be reproductively active later in the season [15]. However, protandrous arrival may not always be beneficial, as its fitness advantages depend on factors such as female availability, the number of competitors present or post-arrival mortality [23].

Body size in salmonids is also an example of a trait that is acted upon by selection and might be linked to fitness [18-20,24-26]. Male salmon exhibit high variability in size at maturity, and such variation results in several alternative tactics to achieve fertilization [15,24,27-31]. Age at maturity is also linked to size variation; smaller, younger-maturing males may adopt sneaking to gain access to spawning females [24,27,28]. On the other hand, large, late-maturating males may engage in fighting and outcompete smaller males to gain access to spawning females [24,27]. In female salmon, variability in size and age tends to be smaller than in males, and studies suggest that large body size may increase their reproductive success [24,32-42].

Within the salmonids, coho salmon (*Oncorhynchus kisutch*) provides a simple study subject for studies on the effect of selection because it is strictly anadromous, and has shorter generation time and simpler age structure compared to most other salmonids; this species typically returns from the ocean at three years of age, while some younger-maturing males, “jacks,” return at two years [15]. Like most *Oncorhynchus*, this species is semelparous [15]; thus, there are no repeat spawners that may hinder precise separation of parental and offspring generations, which could further complicate the assessment of the magnitude and mode of selection based on estimates of individual reproductive success.

The aim of this study was to investigate the temporal variation in selection by comparing the mode, direction and strength of selection on fitness related traits between two cohorts of wild coho salmon. Here, we followed the convention of Siepielski *et al.*[1] and defined the dynamics of temporal variation in selection as “the interannual differences in selection on a given trait within a population.” Specifically, we examined selection on date of return and body length in the wild population at Big Beef Creek, Washington. Information obtained was used to illustrate how selection operates in nature and to provide insights into the temporal dynamics of selection. Pedigree reconstruction based on 11 highly polymorphic DNA microsatellite loci was conducted using more than 3000 individuals, and lifetime reproductive success of two parental brood years was quantified. Younger- and older-maturing males of this species exhibit alternative tactics to maximize reproductive success (sneaking versus fighting). Therefore, the mode, direction and strength of selection acting on males of two age classes and females of a single age class were estimated separately. Estimated selection in this study are the results of both natural and sexual selection, as selection estimates were obtained from regression analyses with lifetime reproductive success as a fitness measure.

## Results

### Characteristics of sampled fish

A total of 3512 returning adults were sampled from 2006 to 2010. On December 3^rd^ in 2007, an atypically large flood breached the weir, possibly allowing some late returning adults to enter the stream unsampled. A total of 1678 individuals were sampled in 2006 and 2007; these were considered candidate parents. In 2008 to 2010, 1834 individuals were sampled; 1240 individuals were likely to be the offspring of the individuals collected in 2006 and 2007, as 3-year-old individuals sampled in 2008 (n = 423) and 2-year-old individuals sampled in 2010 (n = 171) were candidate offspring of individuals returning in 2005 and 2008, respectively. However, all individuals collected were genotyped and included in the analyses.

The number of fish returning in 2007 (n = 1177) was greater than that in 2006 (n = 501; Table 1). The sex ratio (male: female) was greater in 2006 (1.2) than in 2007 (0.8; Table 1). There were consistently more males present in 2006, whereas the male to female sex ratio remained low for the majority of the season in 2007 (Additional file 1). In 2006, the proportion of 2- to 3-year-old males was 0.6:1, but this ratio decreased to 0.06:1 in 2007 (Table 1). The date of return differed between the two years; individuals arrived from November 2^nd^ to December 15^th^ in 2006, but arrived earlier from October 1^st^ to November 20^th^ in 2007. The mean date of return by sex ranged from November 6^th^ (males) to 9^th^ (females) in 2006, and from October 23^rd^ (males) to 26^th^ (females) in 2007 (Table 1). Individuals tended to be smaller in 2007 (Table 1).

**Table 1:** Summary of phenotypic information and reproductive success

**Year**	**Sex**	**Age**	**N**	**Sex Ratio**_**male/female**_	**Ratio**_**2-year-old/3-year-old male**_	**Mean calendar day (days)**	**Mean length (cm)**	**Mean RS**	**Variance RS**
2006	Male	2	104	1.17	0.63	310 (6.20)	34.15 (3.60)	0.69	3.75
	Male	3	166			312 (7.93)	66.57 (7.51)	2.69	20.18
	Female	3	231		NA	313 (10.58)	65.48 (5.40)	2.31	14.98
2007	Male	2	30	0.83	0.06	299 (10.26)	31.56 (3.70)	0.13	0.12
	Male	3	504			296 (11.19)	58.88 (7.66)	0.11	0.17
	Female	3	643		NA	299 (11.23)	59.20 (5.50)	0.10	0.15

Number of individuals (N), sex ratio, ratio of 2-year-old to 3-year-old males, date of return, body length, reproductive success (RS). One standard deviation is shown in parentheses.

### Population genetic statistics

Genotyping error rate was small, with the error rate per locus ranging from 0 to 1.0% (Additional file 2). Across years, no consistent presence of null alleles was detected in all loci, and no large allele dropout or accidental scoring of stuttering were detected. 99.1% of the collected samples (3481 individuals) were successfully genotyped at more than 10 loci, and 99.8% of the collected samples (3507 individuals) were successfully genotyped at more than 6 loci. All loci were moderately to highly polymorphic, with the number of alleles ranging from 9 to 45 and observed heterozygosity ranging from 0.73 to 0.96 (Additional file 3). Among 55 tests (11 loci in each year from 2006–2010), significant deviation from Hardy-Weinberg equilibrium was observed in 25 tests (Additional file 3). Such results may be due to the large sample sizes in 2007 and 2009; however, observed and expected heterozygosity was similar across all loci in all years (Additional file 3). *F*_*IS*_ values tended to be small, ranging from −0.03 to 0.07 across all loci in all years (Additional file 3).

### Parentage analysis

The exclusion probabilities for two-parent and single-parent assignments were > 0.99999 according to FRANz, indicating that the microsatellite dataset provided sufficient power to perform parentage analyses. Results from the tests on the error in our parentage assignment suggest that our error in assigning parents was between 1.7% and 3.6%. Specifically, when pedigree reconstruction was performed with individuals sampled in 2006 as candidate parents and individuals sampled in 2007 as candidate offspring, 3.6% of the assignments (84 out of 2354 assignments) calculated by FRANz had greater than 99% posterior probability. When pedigree reconstruction was performed with individuals sampled in 2007 as candidate parents and individuals sampled in 2006 as candidate offspring, 3.3% of the assignments (33 out of 1002 assignments) calculated by FRANz had more than 99% posterior probability. Assignments with more than 99% probability obtained by FRANz were compared with assignments obtained by COLONY for the 2006 brood year, and 1.7% of mismatches in assignments were observed (25 out of 1490 assignments).

Among 980 returning individuals that could be the offspring of adults returning in 2006, 470 individuals (48.0%) were assigned to both parents, 47 individuals were assigned to a father only (4.8%), 33 individuals were assigned to a mother only (3.4%), and 430 individuals (43.9%) were not assigned to any parents (Table 2). Among 251 returning individuals that could be the offspring of adults returning in 2007, 46 individuals (18.3%) were assigned to both parents, 14 individuals were assigned to a father only (5.6%), 5 individuals were assigned to a mother only (2.0%), and 186 individuals (74.1%) were not assigned to any parents (Table 2). Among a total of 516 individuals that had both parents assigned, 7 individuals were assigned to parents that were sampled across different years (in 2006 or 2007). Given that such matings were impossible, further analyses were performed with and without these assignments. Because the results and their significance did not differ, these assignments were excluded from further analyses.

**Table 2:** Summary of the parentage analysis

**Year**	**Both Parents**	**Father Only**	**Mother Only**	**No Parents**	**Total**
2006	470	47	33	430	980
2007	46	14	5	186	251

Number of offspring that were assigned to both parents, to a father only, to a mother only, or to no parents for the 2006 and 2007 cohort.

**Figure 1:**
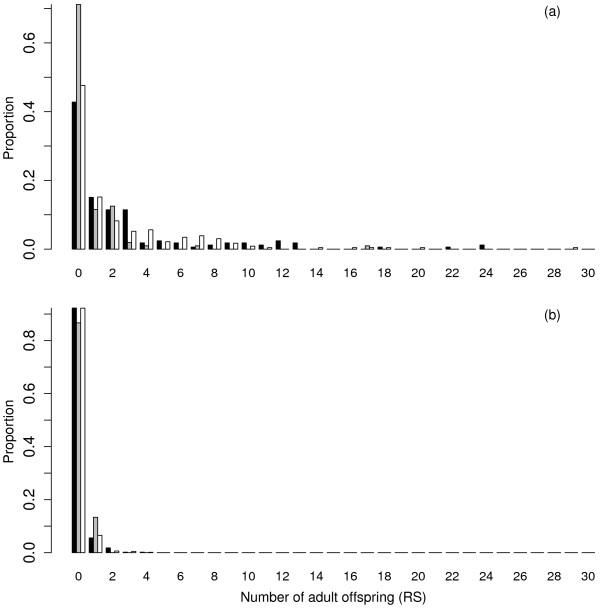
**Reproductive success (RS) of (a) 2006 and (b) 2007 parental cohorts of coho salmon.** The number of adult offspring produced by 3-year-old males (black), 2-year old males (gray) and females (white). Results are displayed as the proportion of parents producing a given number of adult offspring.

The majority of the population produced no or few offspring in both brood years, while some individuals produced a large number of offspring (Figure 1). In 2006, 71.2% of 2-year-old males, 42.8% of 3-year-old males and 47.6% of females produced no returning adult offspring. In 2007, 86.7% of 2-year-old males, 92.3% of 3-year-old males and 92.2% of females produced no returning adult offspring. Average reproductive success in all three groups was higher for the 2006 brood year than the 2007 brood year (Table 1). However, a significant difference in reproductive success between 2006 and 2007 was only detected in 3-year-old males and females (3-year-old males; Mann–Whitney *U*-test, *P* < 0.001; females; Mann–Whitney *U*-test, *P* < 0.001), and no such difference was detected in 2-year-old males (Mann–Whitney *U*-test, *P* = 0.052).

### Selection analysis

#### Date of return

**Figure 2:**
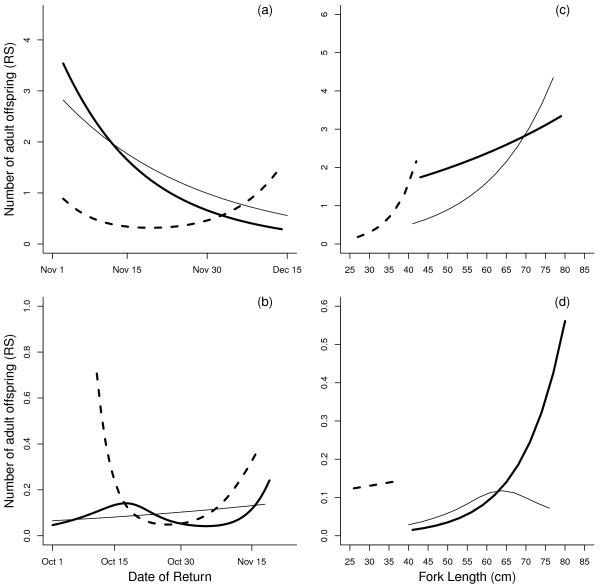
**Relationship between date of return (a, b) or fork length (c, d) and reproductive success.** Plot (a) and (c) are for the 2006 parental cohort, and plot (b) and (d) are for the 2007 parental cohort. Observed data are shown as thick lines for 3-year-old males, thick dashed lines for 2-year-old males and thin lines for females. Note the different scales on *y* axes.

In the 2006 brood year, a positive quadratic selection gradient (*P* < 0.001) on return timing was observed in 2-year-old males, indicating that disruptive selection favored both early and late returning individuals (Table 3); the univariate cubic spline also supported this finding (Figure 2a). A linear gradient in 3-year-old males was negative (*P* < 0.01), indicating that earlier returning individuals had greater reproductive success (Table 3). This trend is also illustrated by the univariate cubic splines (Figure 2a). Neither the linear nor quadratic gradients were significant in females (Table 3), although the cubic spline showed that early returning individuals tended to produce more offspring (Figure 2a).

**Table 3:** Selection gradients for body length and date of return

**Year**	**Sex**	**Age**	**N**	**Intercept**	**Date**	**Length**	**Date**^**2**^	**Length**^**2**^	Date*Length
2006	Male	2	104	-0.57* (0.29)	-0.77 (0.53)	-0.29 (0.32)	0.77*** (0.20)	0.27 (0.20)	-3.23*** (0.92)
	Male	3	166	-0.03 (0.24)	-0.50** (0.16)	NA	NA	NA	NA
	Female	3	231	0.34 (0.21)	-0.10 (0.36)	0.30^*^ (0.12)	-0.12 (0.14)	NA	-0.14 (0.15)
2007	Male	2	30	0.14 (0.50)	NA	-0.12 (0.58)	NA	NA	NA
	Male	3	504	0.01 (0.25)	-0.25 (0.28)	0.83*** (0.24)	NA	-0.12 (0.15)	0.19 (0.18)
	Female	3	643	2.12** (0.76)	0.32 (0.25)	-0.12 (0.27)	-0.53* (0.23)	-0.63** (0.23)	-0.09 (0.30)

*** *P* < 0.001, ** *P* < 0.01, * *P* < 0.05

Coefficients of the best models of relative fitness for the 2006 and 2007 parental cohort. Models were chosen based on the Akaike information criterion (AIC) score. Because of the log-link used with the zero-inflated model, these coefficients are in log space and quadratic terms are not transformed (doubled). One standard deviation is shown in parentheses.

In the 2007 brood year, there was less evidence of selection on return timing. No statistical support for selection on date of return was observed in males of either age class (Table 3). No strong relationship was detected via univariate cubic splines in 3-year-old males. However, the cubic spline implied that both early and late returning individuals tended to produce more offspring for 2-year-old males (Figure 2b). The pattern for 2-year-old males may reflect the lack of power to detect selection in this dataset, as 2-year-old males were rare in 2007 (104 individuals in 2006, 30 individuals in 2007). In females, a significant negative quadratic gradient (*P* < 0.05) was observed, indicating that females returning in the middle of the season had higher fitness than early or late returning females (Table 3). However, no strong relationship was detected via univariate cubic splines in females (Figure 2b).

#### Body Length

In the 2006 brood year, no significant support for selection was detected for body length in males of either age class (Table 3). However, the univariate cubic spline showed that large individuals tended to produce more offspring in both groups (Figure 2c). A significant negative bivariate selection gradient on date of return and length (*P* < 0.001) for 2-year-old males (Table 3) indicated that higher reproductive success of younger- or late-returning males depended on their size. No significant gradient was detected for older males or for females. For females, the linear gradient on length was positive (*P* < 0.05; Table 3), indicating that directional selection favored larger individuals. Graphical representation of the relationship between body length and reproductive success also supported the trend that larger females tended to produce more offspring (Figure 2c).

In the 2007 brood year, no significant selection gradient on length was detected in 2-year-old males (Table 3), and no trend was observed via univariate cubic splines (Figure 2d). In 3-year-old males, selection favored large size, as the linear gradient was positive (*P* < 0.001; Table 3); the cubic splines also showed that larger individuals tended to produce more offspring in this group (Figure 2d). The quadratic gradient in females was negative (*P* < 0.01), indicating that intermediate-sized females had higher fitness than bigger-sized or smaller-sized females (Table 3). The univariate cubic spline also implied that intermediate-sized individuals tended to produce more offspring (Figure 2d).

### Environmental influence on return date

**Figure 3:**
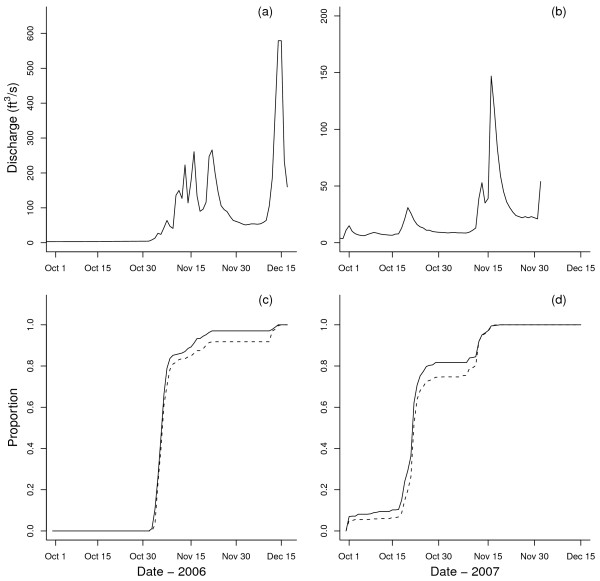
**The amount of water discharge (a, b) and the proportion of arrived spawners (c, d).** Plot (a) and (c) are for 2006, and plot (b) and (d) are for 2007. For the proportion of arrived spawners, observed data are shown as solid lines for males and dashed lines for females. In 2007, discharge data was unavailable from December 3^rd^ to December 15^th^. Note the different scales on stream discharge.

In 2006, the majority of the population returned at the beginning of the run (Figure 3c). In contrast, fish in 2007 returned episodically over a series of peaks throughout the run (Figure 3d). A significant correlation was found between count of daily arrivals and the amount of daily water discharge for both males and females in 2007 (males, ρ = 0.31, *P* < 0.05; females, ρ = 0.30, *P* < 0.05). No such correlation was detected in in 2006.

## Discussion

The aim of our study was to determine the temporal variation in the mode, magnitude and direction of selection in a natural coho salmon population using measures of individual fitness. Our results have shown that reproductive success, as well as selection on body size and date of return to the spawning ground differed markedly between two parental cohorts. Adults in the 2006 brood year had higher reproductive success than those in 2007, and direction and strength of selection differed for both traits between two cohorts. In the first parental cohort, there was significant selection on date of return for 2- and 3-year-old males (disruptive selection on return timing and directional selection favoring earlier return, respectively), and on body length for females (directional selection favoring larger size). In the second parental cohort, there was significant directional selection favoring larger size in 3-year-old males and stabilizing selection on both date of return and size in females. The only evidence for bivariate selection on timing and size was in 2-year-old males in 2006. Observed fluctuations in selection may be due to factors such as changes in precipitation, proportion of 2-year-old versus 3-year-old males, sex ratio and spawner density, as well as the occurrence of catastrophic events (flooding). Although stream discharge itself did not explain variation in relative fitness in all groups, results suggest that precipitation affects return timing of Big Beef Creek coho in some years, potentially explaining the variation in the intensity and the direction of selection on this trait.

Pedigree quality and incorrect assignments can affect the estimated reproductive success and selection gradients [43]. Calculated exclusion probabilities showed that our microsatellite dataset was sufficient for identifying parents, and the addition of more markers is less likely to improve our estimates. Low error rates in our parentage assignment affirmed that microsatellites used in our analyses provided enough statistical power to exclude non-candidate parents. Nevertheless, our overall assignment rate of offspring to one parent or both parents was approximately 50% in the first cohort and 25% in the second cohort. Such results may have occurred due to genotyping errors, to unsampled parents in the candidate parent generations, or to immigration from a non-natal stream during the candidate offspring generations. It is possible that a large number of parents were not sampled at the weir, especially in 2007 when there was a late flood, and this could have led to reduction in assignment success. However, traps were maintained well past the return season and flooding occurred after this period. Additionally, if the number of unsampled parents returned randomly during the season, we would expect to see a larger number of assignments to single parents than to both parents, especially given the number of individuals with unassigned parents. This outcome was not the case. Assignment to single parents varied between two to six percent, and these figures are comparable between both cohorts. While we cannot rule out inefficiency in trapping, especially in 2007, the most plausible explanation for unassigned fish in the offspring generations is that they were immigrants. Parentage estimates in similar systems [17,18,26] acknowledged that not all parents were sampled. In these studies, there was a greater proportion of single parent assignments (32 to 42%) than we observed in this study. All information combined indicates that our sampling was not biased, and the results obtained here were sufficient to gain insights into the factors associated with reproductive success and the process of natural selection on adult size and return timing. Because the estimates of selection were calculated on the parental generation using individual reproductive success, the rate of immigrants in the offspring generation has no bearing on selection estimates. However, we do acknowledge that offspring of the adults that might have emigrated to neighboring streams were not measured in our study, and we might have underestimated individual reproductive success. We assumed that offspring straying was not affected by parental phenotype.

In both 2006 and 2007, the majority of the fish did not produce any returning adult offspring. However, overall reproductive success of the 2006 parental cohort was higher than the 2007 parental cohort across both sexes and age groups. Such a drastic decrease in reproductive success may be explained by the substantial autumn flood that occurred in late 2007. In this year, all individuals arrived by November 20^th^, therefore nests of all females were susceptible to disturbance from the flood that occurred on December 3^rd^. This large flood likely scoured egg pockets of the nests, destroying fish eggs during incubation [44]. A decrease in reproductive success may also be explained by density dependent effects; there was a greater number of spawners present in 2007. Long-term data from Big Beef Creek coho salmon has shown that there is an inverse relationship between the number of female spawners and the number of coho smolts produced by each female [45]. This relationship is likely due to overspawning mortality because greater density of female spawners results in nest destruction and use of less suitable habitat [46-48].

The direction and strength of selection differed for both traits across all groups between the two parental brood years, and these results might be attributable to variation in return numbers, sex ratios and differences in environment. In 2006, precipitation did not occur until the beginning of November, delaying the return timing of the population (Figure 3c). Over 80% of the population returned in the first seven days of the return season, and the male–female sex ratio was particularly high at the beginning of the season (Additional file 1). Selection favoring early return in 3-year-old males suggests that earlier returning individuals may have had increased opportunities for mating under these circumstances [15,22]. Changes in male to female sex ratio during the season revealed that there were consistently more males present; an absence of selection in females thus implies that they were able to secure high-quality sites for nests and obtain mates throughout the spawning season. Interestingly, males of both age classes returned from November 2^nd^ to 22^nd^ and from December 12^th^ to 14^th^. These were the periods of high density in the stream, possibly explaining significant disruptive selection on date of return in 2-year-old males. As the ability of older, larger males to monopolize access to females tends to decrease at higher densities of spawners [49], “sneaking tactics” employed by 2-year-old males were likely more effective during these periods.

In 2007, the return period was protracted, and individuals returned earlier over a series of peaks from October 1^st^ to November 20^th^ (Figure 3d). The male to female sex ratio remained low for the majority of the season (Additional file 1), therefore earlier return might not have been necessary for males to maximize mating opportunities. In females, however, the low male to female sex ratio and the high number of returning females suggest that the opportunity for intrasexual competition among females would have been high. Therefore, return in mid-season may have been important to both secure high-quality sites for nests and gain mates.

Mating success of male salmon is often determined by female choice and intrasexual competition, and studies suggest that there is a positive relationship between male social status and body length [15,50,51]. Unlike in 2006, males were more abundant in 2007; therefore, intrasexual competition among males in 2007 might have resulted in directional selection, favoring larger size in 3-year-old males of this cohort. Although no selection on size was detected in 2-year-old males in 2007, this result may be due to a lack of power because these males were rarer in 2007. Mating success of small, younger-maturing males (“jacks”) is frequency-dependent relative to large, older-maturing males [15,27], and jacks enjoy reproductive advantages when their form is rarer [15,24,27,29-31]. Therefore, it is possible that 2-year-old males had reproductive advantages in 2007, as they were uncommon (39% of all males in 2006; 6% of all males in 2007). In females, selection favored large size in 2006 but intermediate size in 2007. These results were unexpected, as numerous reproductive advantages are known to accompany greater size [16,24,32-42]. However, several studies have suggested that selection may not always favor large size, because efforts of attaining large size also increase the cost associated with growth rate [18,52,53].

Observed patterns of selection can be compared to other studies on natural populations of steelhead trout (19 cohorts, [18]), coho salmon (two cohorts, [19]), and a colonizing population of coho salmon (three cohorts, [20]). All studies estimated selection on body length and return date in males and females. Consistent directional selection toward later return date was observed by Ford *et al.*[19]. In contrast, our findings support the results of Seamons *et al.*[18] and Anderson *et al.*[20], who observed fluctuation in the mode, direction and strength of selection on return date in both sexes. Variation in selection might have been observed because of the large number of replicates in Seamons *et al.*[18] and Anderson *et al.*[20]. As we observed, environmental conditions (e.g., precipitation) likely affect return date in salmon; thus, accurate patterns of selection on this trait may only be obtained using sufficient replicates. Anderson *et al.*[20] observed consistent directional selection on body length, favoring large size in both sexes. In contrast, and similar to our study, Seamons *et al.*[18] and Ford *et al.*[19] observed fluctuations in the mode and direction of selection on this trait in both sexes. Such results may reflect temporal variation in intraspecific competition. In particular, Ford *et al.*[19] observed changing proportions of younger- and older-maturing males between two cohorts; different frequencies of these males potentially resulted in variation in intensity of intraspecific competition, leading to fluctuating selection. Additionally, Seamons *et al.*[18] attributed changes in sex ratio and breeding density in both sexes as a potential cause of fluctuating selection. Our findings support both studies because changes in the proportion of younger- and older-maturing males, sex ratio and breeding density likely caused fluctuations in selection.

Because coho salmon at Big Beef Creek is a long established population, the distribution of studied traits was presumed to be at a stable optimum, and weak selection was expected. However, strong selection was observed in some years, and the direction and strength of selection were not consistent between two cohorts in this study. The extensive review on selection in natural populations found that the direction and strength of selection vary substantially and that quadratic selection is typically quite weak in wild [2,5]. Established wild populations may be “chasing” fluctuating optima, and that selection can be quite strong in some years, and not so in others, and that the direction of selection varies over time [1]. Such fluctuations in selection may act to maintain phenotypic variation in the traits in question, and we may have observed this process in our study.

## Conclusions

We found evidence of selection on body length and date of return to the spawning ground, both of which are important fitness-related traits in salmonids. Reproductive success and the mode, direction and strength of selection widely varied between two parental brood years; differences may be due to factors such as annual changes in precipitation, occurrence of catastrophic events (flooding), the proportion of younger- versus older-maturing males, sex ratio and densities of spawners. As these factors are seldom consistent each year, long-term studies may be important to gain insights into anticipated evolutionary change.

## Methods

### Study Area and sampling

This study was conducted at Big Beef Creek (47°39’N, 122°46’W) in Washington State, USA, situated on Hood Canal in Puget Sound. Big Beef Creek is routinely monitored as an indicator stream for long-term ecological studies in the region, and the amount of water discharge (cubic feet per second) from the creek is measured on a daily basis [54].

The creek supports a healthy native run of anadromous coho salmon, which is indigenous to the system and has been monitored by the Washington Department of Fish and Wildlife (WDFW) over the past 30 years. As hatchery practices have never been conducted at this creek, we expect no or little effects on the current genetic structure of the population. A weir is placed at the mouth of the creek, and only naturally spawned fish (all hatchery fish are externally marked) are passed over the weir for subsequent spawning. Therefore, intrusion of hatchery fish into the wild population at this creek has been prevented as far as possible.

Returning adults of coho salmon were sampled every year from 2006 to 2010. Records indicate that peak river entry of coho salmon to this creek occurs from late-October to mid-November [55]. The brood class of this species returns on average at 3 years, but early maturing males return at 2 years; therefore, sampling provided 2 full cohorts, for parental brood years 2006 and 2007 and their adult offspring returning in 2008 to 2010.

As each individual passed the weir, date of return, body length (length from the tip of snout to fork of tail) and sex were recorded, and a fin clip was obtained for DNA analysis. In coho salmon, returning males smaller than 35cm in body length are typically 2 years old, and returning males larger than 45cm are 3 years old [45]. Scales from all individuals ranging between 35cm and 45cm were collected and read by WDFW to verify exact age. Once measurements were taken, individuals were allowed to swim upstream for spawning. Data from Big Beef Creek coho suggest that retuning coho spawn for up to three weeks after river entry [45].

This study was reviewed and approved by the University of Washington's Institutional Animal Care and Use Committee (IACUC).

### Microsatellite analysis and parentage

Genomic DNA from sampled individuals was extracted using the DNeasy extraction kit (QIAGEN, Valencia, CA, USA) following the manufacturer’s procedures. Extracted DNA was used to amplify 11 microsatellite loci (Additional file 2) via multiplex Polymerase Chain Reaction (PCR) using a QIAGEN Multiplex PCR kit. Specifically, reaction mixtures consisted of 10-200ng genomic DNA, 1x QIAGEN Multiplex PCR Master Mix, 0.03 μM – 0.4μM of each primer, making up a total volume of 10μl (Additional file 2). Cycling conditions consisted of a 15-min, initial activation step at 95°C, 30 cycles of 30-s denaturing step at 94°C, 90-s annealing step at 57–60 °C and 90-s extension step at 72 °C, and a 30-min, final extension step at 60°C (Additional file 2). The forward primer of every locus was labeled with fluorescent dye. Individuals were genotyped using a 96-capillary system Molecular Dynamics MegaBACE 1000 automatic genotyper (GE Healthcare, Piscataway, NJ, USA), and Genetic Profiler version 2.2 was used to determine fragment sizes of all loci. To calculate our genotyping error rate, 96 individuals from samples obtained in 2006 were randomly chosen, re-extracted and genotyped. The genotyping error rate was estimated by calculating the percentage of allele calls that were different between two analyses.

MICROCHECKER v. 2.2.3 was used to estimate the frequency of null alleles, as well as to screen for large allele dropout and accidental scoring of stuttering [56]. GenePop v. 4.0.10 was used to perform exact tests for deviations from Hardy-Weinberg equilibrium [57]. GenAlEx v.6.41 was used to calculate observed and expected heterozygosities, as well as Weir and Cockerham’s *F*_*IS*_ values [58]. FRANz v. 1.9.999 was used to calculate exclusion probabilities for two-parent and single-parent assignments based on the 11 loci used for the analyses, as well as to perform pedigree reconstruction [59]. This program uses a log-ratio, the parent-pair log-odds ratio (LOD) score to assign parentage, and it estimates statistical confidence for each assignment using Markov Chain Monte Carlo (MCMC) sampling. All adults returned in 2006 and 2007 were considered as candidate parents for individuals returned in 2008 to 2010. Only individuals that were genotyped at more than 6 loci were included in the analysis. Parentage assignments that had a posterior probability higher than 0.99 were used for further analysis.

We performed two tests to assess our assignment error rate. First, FRANz v. 1.9.999 was used to perform pedigree reconstruction with unlikely parent-offspring pairings: 1) individuals sampled in 2006 as candidate parents and individuals sampled in 2007 as candidate offspring, and 2) individuals sampled in 2007 as candidate parents and individuals sampled in 2006 as candidate offspring. Paternity and maternity assignments that had more than 99% posterior probability from these trials were used to calculate our parentage error rate. Second, COLONY v. 2.0 was used to perform pedigree reconstruction for the first parental brood year (2006) and their candidate offspring as a comparison [60-62]. This program implements a maximum likelihood method to assign parentage among individuals using multilocus genotype data, and provides the approximated probability for each assignment. Both sexes were allowed to be polygamous. Only parentage assignments that had probability of more than 0.99 were employed and compared with assignments obtained from FRANz v. 1.9.999 that had probability of more than 0.99. It was not possible to perform a similar comparison on the whole dataset, because COLONY was computationally intensive.

### Selection analysis

Reproductive success (RS), defined as the number of returning adult offspring produced by each parent, was calculated using the estimated parentage assignments, and was used as a measure of fitness for selection analysis. The Mann–Whitney *U*-test was used [63] to test differences in reproductive success of 2-year-old males, 3-year-old males and females between the two parental brood years. Body length and date of return were standardized within each sex, age in males and cohort to a mean of zero and to a standard deviation of one, denoted as *z*_l_ (body length) and *z*_d_ (date of return). Relative fitness, *w*, was calculated for each individual by dividing its reproductive success by the within-sex, within-age (in males) and within-brood year mean [64,65]. Selection analyses were performed using the zero-inflated regression model implemented in the pscl package in R [66], with negative binomial error distribution and the canonical log link. Zero-inflated models describe the data better than the simple generalized linear model, as relative fitness exhibited overdispersion and a large number of true zero values (parents with no returning offspring). In order to impose relative fitness as a response variable, all zero-inflated models in our analysis included an offset term of the logarithm within-sex, within-age and within-brood year mean reproductive success. Regression was performed separately on 2-year-old males, 3-year-old males and females in each brood year. The initial model for all groups included five coefficients $\left(z_{1,}z_{d},z_{\text{l}}{}^{2},z_{d}{{}^{2}}_{,}z_{\text{l}}z_{d}\right)$ for each of the explanatory variables:

(1) $$w=\alpha +z_{\text{l}}+z_{d}+z_{\text{l}}{}^{2}+z_{d}{}^{2}+z_{1}z_{d}$$

*w* is relative fitness, α is the *y*-intercept of the fitness function, and *z*_l_ and *z*_d_ are standardized body length and date of return for each individual, respectively. Originally, stream discharge on each individual’s return date was also included in the initial model, however it was removed because discharge itself did not explain variation in relative fitness in all groups of both cohorts. Final model selection using the Akaike information criterion (AIC) was based on a stepwise method recommended by Zuur *et al.*[67]. Final models were validated by fitting the residuals against each explanatory variable.

Univariate cubic splines were calculated following Schluter [68], in order to visualize the relationship between reproductive success and measured traits in both males and females. Specifically, these analyses were performed using the generalized additive model, GAM function, implemented in R with negative binomial error distribution and an additional overdispersion parameter, θ [69]. θ was calculated for 2-year-old males, 3-year-old males and females within each parental brood year according to the equation:

(2) $$\theta =\frac{mean{\left(RS\right)}^{2}}{\mathrm{var}(RS)-\text{mean}(RS)}$$

The smoothing parameter, λ, was estimated for each curve using the generalized cross-validation (GCV) criterion implemented in the GAM function, available from the mgcv library in R [70].

### Environmental influence on return date

It is known that upstream migration is influenced by precipitation in coho salmon [15]. Spearman’s rank correlation test was performed to investigate the relationship between the number of arrived spawners counted on a daily basis and the amount of daily water discharge in each parental brood year, as neither variable was normally distributed [63].

## Competing interests

The authors declare that they have no competing interests in relation to this manuscript.

## Authors’ contributions

KAN and JJH designed and supervised the study. MK processed the genetic data, performed the analyses and prepared the first draft of the manuscript in partial fulfillment of a graduate degree at the University of Washington. KAN was the PI for the overall project. All authors have read and approved the final manuscript.

## Supplementary Material

Additional file 1 **Changes in male to female sex ratio. Plot (a) is for the 2006 parental cohort, and plot (b) is for the 2007 parental cohort. Note the different scales on *y*axes.** Click here for file

Additional file 2 **Microsatellite loci used for parentage analysis of coho salmon. T**_a_ = annealing temperature. Repeat units and allele ranges are given in base pairs (bp) [71-76]. Click here for file

Additional file 3 **Population genetic data at 11 microsatellite loci in coho salmon samples. **N = number of genotyped inidividuals, N_A_ = number of alleles, H_O_ = observed heterozygosity, H_E_ = expected heterozygosity, P_HWE_ = probability of Hardy-Weinberg equilibrium (significant results in bold), F_IS_ = inbreeding coefficient. Click here for file
