# Supplementary figures and images for: Temporal variation in selection on body length and date of return in a wild population of coho salmon, Oncorhynchus kisutch
Source: BMC Evol Biol. 2012 Jul 17;12:116. doi: 10.1186/1471-2148-12-116 (PMC3482603; doi:10.1186/1471-2148-12-116)

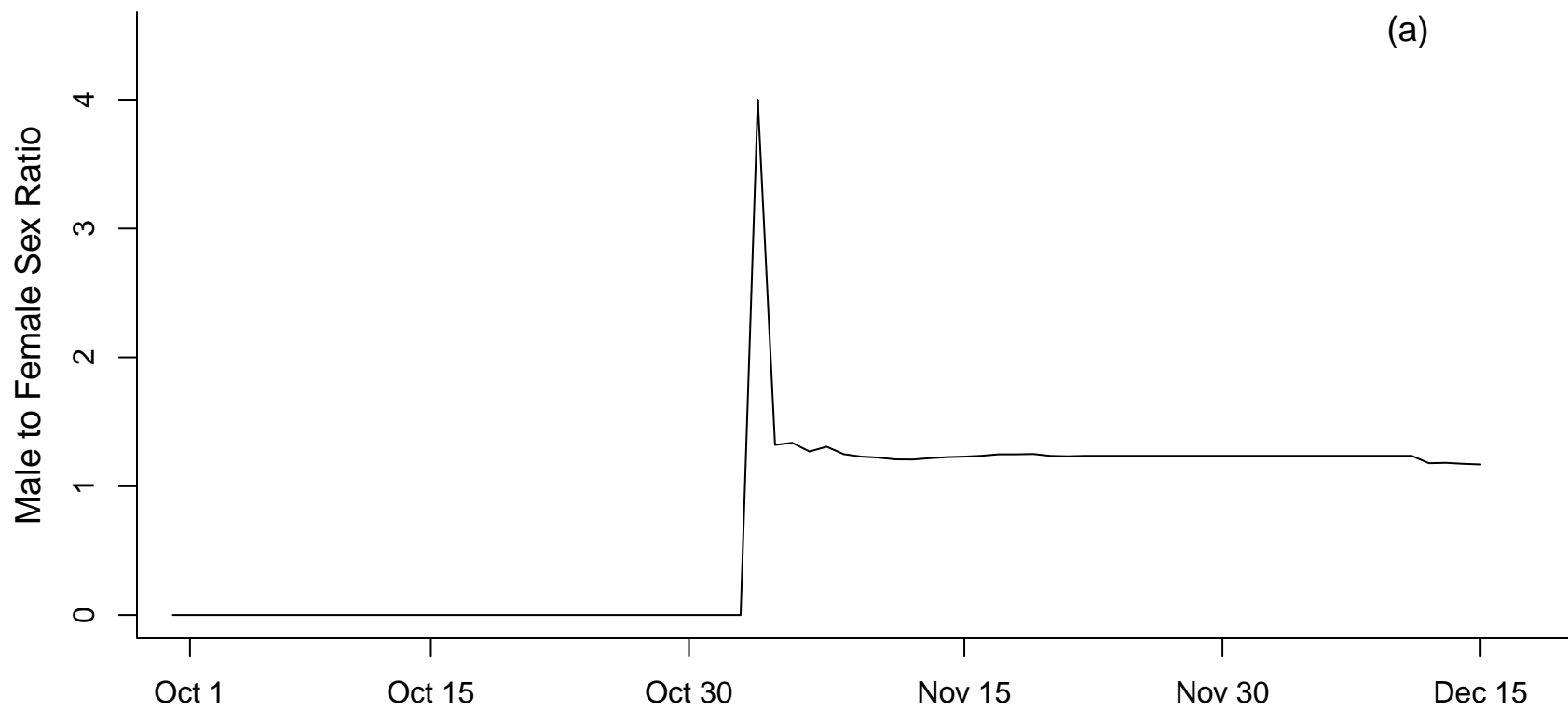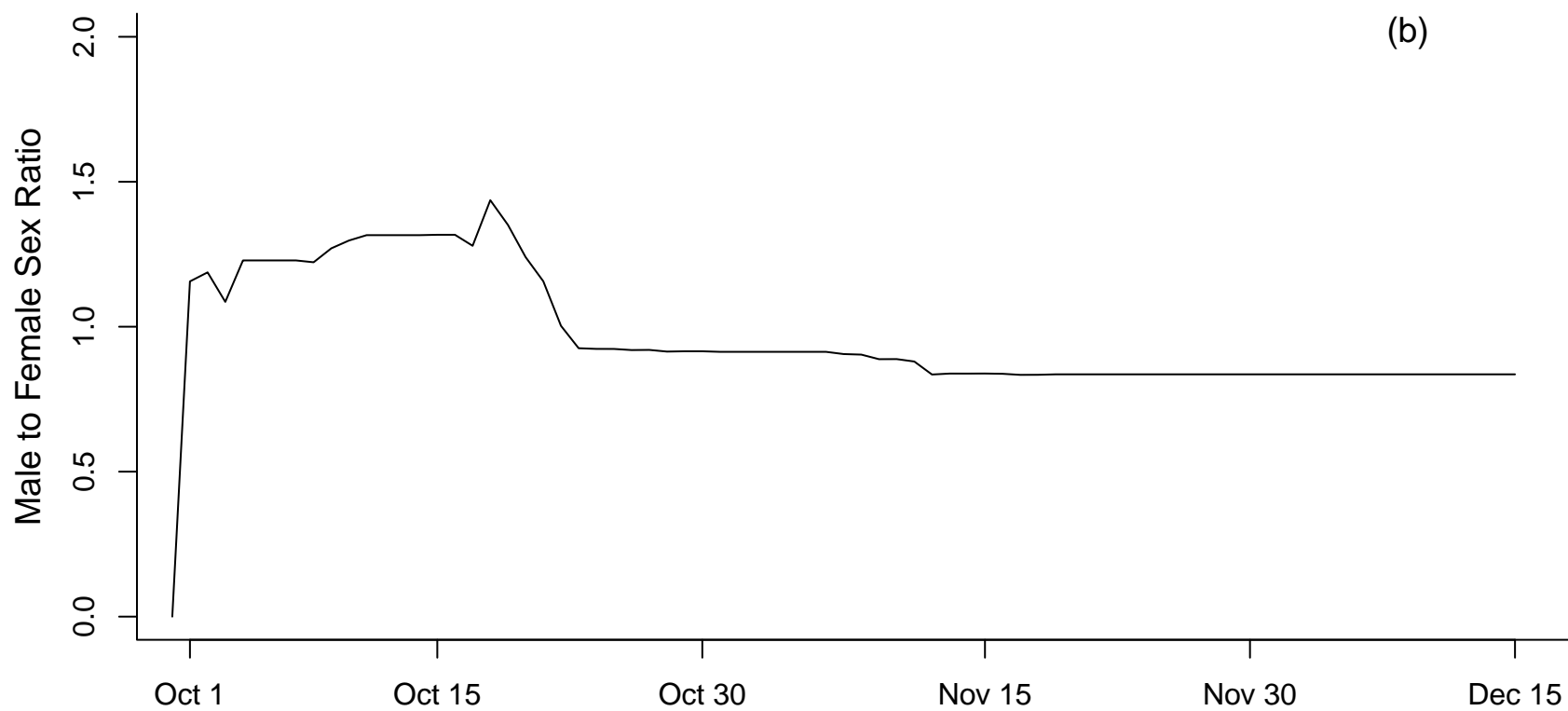

Supplement: Additional file 1 — Changes in male to female sex ratio. Plot (a) is for the 2006 parental cohort, and plot (b) is for the 2007 parental cohort. Note the different scales on yaxes. [file 1471-2148-12-116-S1.pdf]
